# Ex vivo Akt inhibition promotes the generation of potent CD19CAR T cells for adoptive immunotherapy

**DOI:** 10.1186/s40425-017-0227-4

**Published:** 2017-03-21

**Authors:** Ryan Urak, Miriam Walter, Laura Lim, ChingLam W. Wong, Lihua E. Budde, Sandra Thomas, Stephen J. Forman, Xiuli Wang

**Affiliations:** T cell Therapeutics Research Laboratory, Department of Hematology & Hematopoietic Cell Transplantation, City of Hope National Medical Center, 1500 E. Duarte Rd., Duarte, CA 91010 USA

**Keywords:** Akt inhibitor, CAR T cell therapy, B cell malignancies

## Abstract

**Background:**

Insufficient persistence and effector function of chimeric antigen receptor (CAR)-redirected T cells have been challenging issues for adoptive T cell therapy. Generating potent CAR T cells is of increasing importance in the field. Studies have demonstrated the importance of the Akt pathway in the regulation of T cell differentiation and memory formation. We now investigate whether inhibition of Akt signaling during ex vivo expansion of CAR T cells can promote the generation of CAR T cells with enhanced antitumor activity following adoptive therapy in a murine leukemia xenograft model.

**Methods:**

Various T cell subsets including CD8+ T cells, bulk T cells, central memory T cells and naïve/memory T cells were isolated from PBMC of healthy donors, activated with CD3/CD28 beads, and transduced with a lentiviral vector encoding a second-generation CD19CAR containing a CD28 co-stimulatory domain. The transduced CD19CAR T cells were expanded in the presence of IL-2 (50U/mL) and Akt inhibitor (Akti) (1 μM) that were supplemented every other day. Proliferative/expansion potential, phenotypical characteristics and functionality of the propagated CD19CAR T cells were analyzed in vitro and in vivo after 17-21 day ex vivo expansion. Anti-tumor activity was evaluated after adoptive transfer of the CD19CAR T cells into CD19+ tumor-bearing immunodeficient mice. Tumor signals were monitored with biophotonic imaging, and survival rates were analyzed by the end of the experiments.

**Results:**

We found that Akt inhibition did not compromise CD19CAR T cell proliferation and expansion in vitro, independent of the T cell subsets, as comparable CD19CAR T cell expansion was observed after culturing in the presence or absence of Akt inhibitor. Functionally, Akt inhibition did not dampen cell-mediated effector function, while Th1 cytokine production increased. With respect to phenotype, Akti-treated CD19CAR T cells expressed higher levels of CD62L and CD28 as compared to untreated CD19CAR T cells. Once adoptively transferred into CD19+ tumor-bearing mice, Akti treated CD19CAR T cells exhibited more antitumor activity than did untreated CD19CAR T cells.

**Conclusions:**

Inhibition of Akt signaling during ex vivo priming and expansion gives rise to CD19CAR T cell populations that display comparatively higher antitumor activity.

**Electronic supplementary material:**

The online version of this article (doi:10.1186/s40425-017-0227-4) contains supplementary material, which is available to authorized users.

## Background

Adoptive transfer of in vitro-expanded chimeric antigen receptor (CAR) T cells genetically modified to express tumor-targeted antigen receptors can result in dramatic regressions of B cell leukemia [1, 2]. However, insufficient persistence and effector function of CAR-redirected T cells in vivo has been a challenge for adoptive T cell therapy [3–6]. Manufacturing CAR T cells is a process that involves activation and ex vivo culture with IL-2 to facilitate CAR gene transfer and to achieve a therapeutic number of T cells. This process is also associated with decreased antitumor activity due to terminal differentiation [7].

Studies indicate that transfer of potent CAR T cells with central memory traits can enhance antitumor immunity and the curative potential of adoptive therapy for cancer [8–10]. Recent studies have highlighted the importance of the Akt and Wnt pathways in the regulation of CD8+ T cell differentiation and memory formation [11–14]. Upon engagement of cell surface receptors such as the T cell receptor, co-stimulatory molecules, and cytokine receptors, the PI3K-Akt pathway is activated, resulting in downstream responses via phosphorylating a range of intracellular proteins. Akt activation is required for T cell activation [11]. However, studies have shown that the sustained activity of Akt progressively drives T cells toward terminal differentiation and diminished anti-tumor activity [11]. Crompton and van der Waart et al. have shown that reduction of Akt activation during T cell ex vivo expansion resulted in potent tumor-infiltrating lymphocytes (TIL) and T cells specific for minor histocompatibility antigens (MiHAs) on tumors [15, 16]. We therefore hypothesized that manipulation of the magnitude of Akt activation may prevent terminal differentiation of CAR T cells as they are expanded to a therapeutic dose. To evaluate this hypothesis, we transduced and expanded CD19CAR T cells in the presence of an Akt inhibitor. We show that Akt inhibition during ex vivo expansion did not inhibit CD19CAR T cell proliferation and effector function but gave rise to less differentiated CAR T cells with high expression of CD62L and CD28 [7]. Additionally, the CAR T cells expanded in the presence of the Akt inhibitor appeared to have enhanced antitumor activity in vivo. Taken together, these findings suggest the use of pharmacologic approaches during the ex vivo expansion of CAR T cells could achieve favorable attributes of CAR T cells for improved T cell therapy.

## Methods

### Cell lines

Unless stated otherwise, all cell lines were maintained in RPMI 1640 (Irvine Scientific) supplemented with 2 mM L-glutamine, 25 mM HEPES, and 10% heat-inactivated FCS (Hyclone). PBMCs were transformed with Epstein-Barr virus to generate lymphoblastoid cell lines (LCLs) as previously described [17]. To generate OKT3-expressing LCLs (LCL OKT3), allogeneic LCLs were re-suspended in nucleofection solution using the Amaxa Nucleofector kit. OKT3-2A-Hygromycin_pEK plasmid was added to 5 mg/10^7^ cells, the cells were electroporated using Amaxa Nucleofector I, and the resulting cells were grown in RPMI-1640 with 10% FCS containing 0.4 mg/mL hygromycin. To generate firefly luciferase (ffluc) + GFP+ CD19+ lymphoid leukemic cells, SupB15 cells obtained from ATCC were transduced with a lentiviral vector encoding eGFP-ffluc. Initial transduction efficiency was 50%; therefore, the GFP cells were sorted by FACS for >98% purity (fflucGFPSupB15s).

### Antibodies and flow cytometry

Human T cells were analyzed by flow cytometry after staining with fluorochrome-conjugated monoclonal antibodies (mAbs) to CD8 (SKI), CD62L (Dreg-56), CD45RO (UCHL1), CD45RA (L48), CD28 (CD28.2), CD3 (SK7), IFN-γ (XMG1.2), CD107a (H4A3), KLRG1 (2 F1/KLRG1), CD57 (HNK-1), Tim3 (F38-2E2), PD1 (EH12.1), LAG3 (FAB2319C), and phosphorylated Akt (pAkt, pS473)(M89-61) (BD Biosciences). Data acquisition was performed on a MACSQuant (Miltenyi Biotec) and analyzed using FCS Express, Version 3 software (De Novo Software). Telomere length analysis was performed using the flow-FISH technique with Telomere PNA Kit/FITC obtained from Dako (Dako Denmark A/S, Denmark). Biotinylated Erbitux (cetuximab) and streptavidin-PE were used to identify T cells that expressed truncated human EGFR (EGFRt). Anti-CD4 microbeads were purchased from Stemcell Technologies, and anti-CD14, anti-CD45RA, and anti-CD25 microbeads were purchased from Miltenyi Biotec. Proliferation capacity was determined by labeling the cells with carboxyfluorescein succinimidyl ester (CFSE) (Invitrogen).

### Generation of CD19 CAR T cells

For isolation of CD8+ T cells, CD4+ T cells in PBMCs were labeled with anti-CD4 microbeads and depleted with the EasySep system (Stemcell Technologies). The freshly isolated CD4 negative cells were activated with CD3/CD28 Dynabeads (Invitrogen) and transduced the next day with a lentivirus encoding CD19R (EQ):CD28:ζ/EGFRt at an MOI of 3 and expanded in the presence of 50U/mL rhIL-2 (CellGenix) and 1 μM Akt inhibitor VIII (Akti-1/2) (Merck Millipore). Cultures were then supplemented with 50 U/mL rhIL-2 (CellGenix) and Akt inhibitor every 48 h for 17–21 days before in vitro analysis and adoptive transfer. Transduced CD19CAR T cells without Akt inhibitor treatment were used as control, as were non-transduced mock cells from the same donor. In some experiments, bulk T cells, purified CD62L + CD45RO+ central memory (T_CM_) and CD62L+ naïve/memory T cells were engineered with CD19CAR using the selection platform that we have developed [18]. Briefly, for T_CM_ isolation, fresh PBMC were incubated with anti-CD14, anti-CD45RA, and anti-CD25 microbeads, and the resulting labeled cells were then immediately depleted using the CliniMACS™ depletion mode to remove CD14+ monocytes, CD25 + Treg T cells and CD45RA+ naïve T cells. The resulting unlabeled negative fraction cells were then labeled with biotinylated-anti-CD62L (DREG56) mAb and anti-biotin microbeads. The CD62L + CD45RO + T_CM_ were purified with positive selection. Similarly, fresh PBMC were incubated with anti-CD14 and anti-CD25 microbeads, and the resulting labeled cells were then immediately depleted using the CliniMACS™ to remove CD14+ monocytes and CD25 + Treg T cells. The resulting unlabeled negative fraction cells were then labeled with biotinylated-anti-CD62L mAb and anti-biotin microbeads. The CD62L^+^ naïve/memory T cells were purified with positive selection.

### DNA constructs

The lentivirus CAR construct was modified from the previously described CD19-specific scFvFc:ζ chimeric immunoreceptor [19] to create a second-generation vector. The CD19CAR containing a CD28z costimulatory domain carries mutations at two sites (L235E; N297Q) within the CH2 region on the IgG4-Fc spacers to ensure enhanced potency and persistence after adoptive transfer [20]. The lentiviral vector also expresses a truncated human epidermal growth factor receptor (huEGFRt), which includes a cetuximab (Erbitux) binding domain but excludes the EGF-ligand binding and cytoplasmic signaling domains. A T2A ribosome skip sequence links the codon-optimized CD19R:CD28:ζ sequence to the huEGFRt sequence, resulting in coordinate expression of both CD19R:CD28:ζ and EGFRt from a single transcript [21]. The CD19CARCD28EGFRt DNA sequence (optimized by GeneArt) was then cloned into a self-inactivating (SIN) lentiviral vector, pHIV7 (gift from Jiing-Kuan Yee, Beckman Research Institute of City of Hope, Duarte, CA) (Additional file 1: Figure S1).

### CFSE proliferation

Ex vivo expanded CD8 + CD19CAR T cells with and without Akt inhibitor treatment were labeled with 0.5 mM CFSE and co-cultured with CD19+ LCLs as stimulator cells for 5 days in the presence of 50U/L rhIL-2. CFSE dilution of CD3 and CAR double positive populations was determined using multicolor flow cytometry. In some experiments, CD19CAR T cells derived from PBMC and T_CM_ were used for proliferation assays.

### Degranulation and cytokine production assays

Ex vivo expanded CD8 + CD19CAR T cells (10^5^) with and without Akt inhibitor treatment were co-cultured with LCLs (10^5^) in medium containing Golgi plug (BD) and antibody against CD107a for six hours at 37 °C. Acute myeloid leukemia KG1a cells were used as a negative stimulator and LCL OKT3 as positive stimulator. Degranulation was determined using multicolor flow cytometry. Ex vivo expanded CD8 + CD19CAR T cells with and without Akt inhibitor treatment (10^5^) were co-cultured in 96-well tissue culture plates with 10^5^ of the indicated stimulator cells. Supernatants were harvested 18 h after stimulation, and cytokines were measured by a cytometric bead array using the Bio-Plex Human Cytokine Panel (Bio-Rad Laboratories) (in triplicates) according to the manufacturer’s instructions.

### Xenograft model

To establish a leukemic model, acute lymphoid leukemic SupB15 cells were engineered with GFP firefly luciferase (GFPffluc+), and 0.5 × 10^6^ purified GFP+ cells were inoculated into six- to ten-week old NOD/*Scid* IL-2RγC^null^ (NSG) mice intravenously (i.v) on day -5. After confirmation of the tumor engraftment, 1–2 × 10^6^ expanded CD19CAR T cells were adoptively transferred into tumor-bearing mice intravenously. Tumor signals were monitored by Biophotonic tumor imaging.

### Statistical analysis

Analyses were performed using Prism (GraphPad Software Inc.). The Mann-Whitney t- and Log-rank (Mantel-Cox) tests were used to ascertain the statistical significance of the in vivo data (tumor signals and survival). The Wilcoxon matched-pairs signed rank test (2-tailed) was used for the analysis of in vitro data except the cytokine data assumed to have a normal distribution in triplicates were analyzed with paired *t* test. *P* < 0.05 was considered statistically significant.

## Results

### Akt inhibition does not compromise CD19CAR T cell proliferation and expansion

**Fig. 1:**
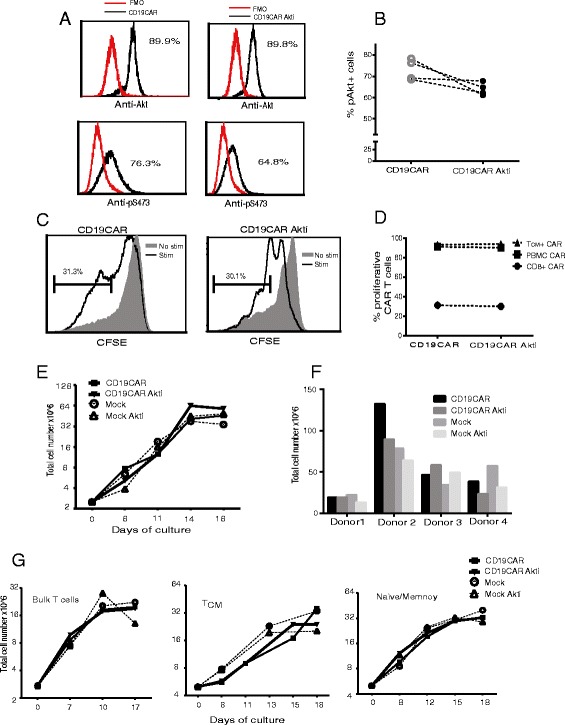
Akt inhibition does not compromise CD19CAR T cell proliferation and expansion. CD8+ T cells in PBMC from healthy donors were isolated by CD4+ T cell depletion following anti-CD4 microbeads labeling and magnetic selection with the EasySep system. The freshly isolated CD4 negative cells were activated with CD3/CD28 Dynabeads and transduced the next day with CD19R (EQ):CD28:ζ/EGFRt lentivirus at an MOI of 3, then expanded. The cultures were supplemented with 50 U/mL rhIL-2 and 1 μM Akt inhibitor every 48 h for 17–21 days before in vitro analysis. Transduced CD19CAR T cells without Akt inhibitor treatment were used as control. **a** Expanded CD8 + CD19CAR T cells were harvested and stained intracellularly with antibodies against phosphorylated Akt pS473 and total Akt. Fluorescence minus one (FMO) are depicted in red histograms. Histograms are of gated CD19CAR+ T cells. **b** Percentages of phosphorylated Akt positive cells from four different donors are presented. **c** Expanded CD8 + CD19 CAR T cells were labeled with 0.5 mM CFSE and then co-cultured with CD19+ LCLs as stimulators for 5 days in the presence of 50U/L rhIL-2. CFSE dilution of gated CD3+ and CAR+ double positive populations was determined using multicolor flow cytometry. Percentages of CFSE negative cells over non-stimulated cells are presented. **d** Percentages of CFSE negative CAR T cells derived from different T cell subsets are depicted. **e** CD8+ T cells were transduced with lentivirus encoding CD19CAR vector and expanded in a medium containing 50U/L rhIL2, in the presence and absence of 1 μM Akt inhibitor. Viable cell numbers were determined by Guava ViaCount at the indicated time points. **f** Data on day 17 from four different donors are presented. **g** Bulk T cells, purified T_CM_ and purified naïve/memory T cells were transduced with lentivirus encoding second generation CD19CAR vector and expanded in a medium containing 50U/L rhIL2, in the presence and absence of 1 μM Akt inhibitor. Viable cell numbers were determined by Guava ViaCount at the indicated time points. Mock T cells from the same donor were used as controls

To investigate the effects of Akt inhibition on CAR T cell expansion and function, we first sought to confirm that the Akt inhibitor (Akti) in culture can reduce phosphorylation of Akt, especially serine residue phosphorylation. Akti VIII, which selectively inhibits Akt1/Akt2 activity, has been shown to be able to induce memory T cell formation at a concentration of 1 μM [16]. We therefore transduced purified CD8+ T cells and expanded CD8 + CD19CAR T cells in the presence of 1 μM Akti. After two to three weeks of ex vivo expansion, intracellular pAkt in the CD8 + CD19CAR T cells were analyzed with flow cytometry. We consistently found that 1 μM Akti resulted in a trend toward modest reduction (73.1 ± 2.5 to 64.0 ± 1.5%) of phosphorylation of Akt on serine 473 in CD19CAR T cells across four different donors, but left total Akt signaling unaltered (*N* = 4, *P* = 0.1) (Fig. 1a and b). We then investigated whether this level of reduction of Akt affects CAR T cell proliferation and expansion. Interestingly, we did not observe an effect of Akt inhibition on proliferation based on the CFSE dilution of CD8+ CD19CAR T cells (Fig. 1c), nor of bulk PBMC and T_CM_ derived CD19CAR T cells (79.1 ± 13.6% of untreated vs. 77.6 ± 14.9% of Akti-treated CD19CAR T cells) (Fig. 1d). Total cell growth was not compromised by inhibition of Akt signaling during 17 days of ex vivo expansion, which is the maximum days of T cell expansion used in our current clinical trials, indicating the intact proliferation and expansion capacity of Akti-treated CD19CAR T cells (Fig. 1e). These data were consistent with CD8+ T cells from multiple donors (Fig. 1f). For most adoptive T cell therapies, CAR T cell products are derived from a mixture of T cell populations containing both CD4+ and CD8+ T cells. We further analyzed the impact of the Akt inhibitor on the CD19CAR T cells containing both CD4+ and CD8+ subsets. Again, proliferation and expansion were not inhibited in this composition of CD19CAR T cells (Fig. 1g). Considering that the activation threshold of various T cell subsets differs [22, 23], a study of the impact of Akti on the various T cell subsets was performed. Purified T_CM_ and naïve/memory T cells were activated, transduced and expanded in the presence of 1 μM Akti. Consistently, we observed no negative effects of Akti on ex vivo expansion of all the T cell subsets tested (Fig. 1g).

### Akt inhibition generates CD19CAR T cell populations with memory-like characteristics

**Fig. 2:**
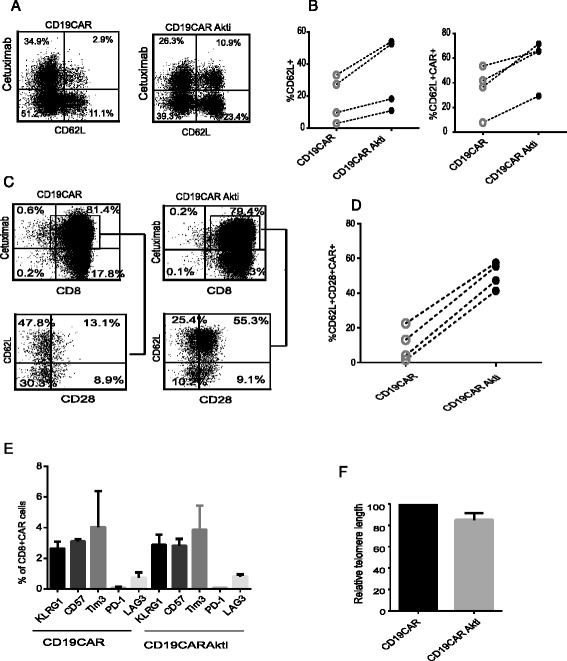
Akt inhibition generates CD8 + CD19CAR T cell populations with memory-like characteristics. CD8 + CD19CAR T cells that had been cultured in the presence and absence of 1 μM Akti were stained with biotinylated Erbitux (cetuximab), followed by streptavidin-PE for CAR detection and antibodies against CD8, CD62L, CD28, KLRG1, CD57, Tim3, PD1, and LAG3. **a** Percentages of CD62L+ and CAR + CD62L+ double positive cells are depicted on the basis of the gating of isotype-stained cells. **b** Percentages of CD62L+ and CAR + CD62L+ double positive cells from four different donors are presented. **c** Expression and percentages of CD62L and CD28 after gating on CAR+ cells are presented. **d** Percentages of CD62L + CD28 + CAR+ cells after gating on CAR+ cells. Data from four different donors are presented. **e** Mean percentages of immunoreactive cells + SEM from 4 different donors are presented. **f** In vitro expanded CD8 + CD19CAR+ T cells were collected. Relative telomere length of T cells was determined with Telomere PNA detection kit and normalized against 1301 cell line as control. Means + SEMs of duplicates from three different donors are depicted. RTL = (mean FL1 sample cells with probe-mean FL1 sample cells without probe) × DNA index of control cells ×100/(mean FL1 control cells with probe-mean FL1 control cells without probe) × DNA index of sample cells

**Fig. 3:**
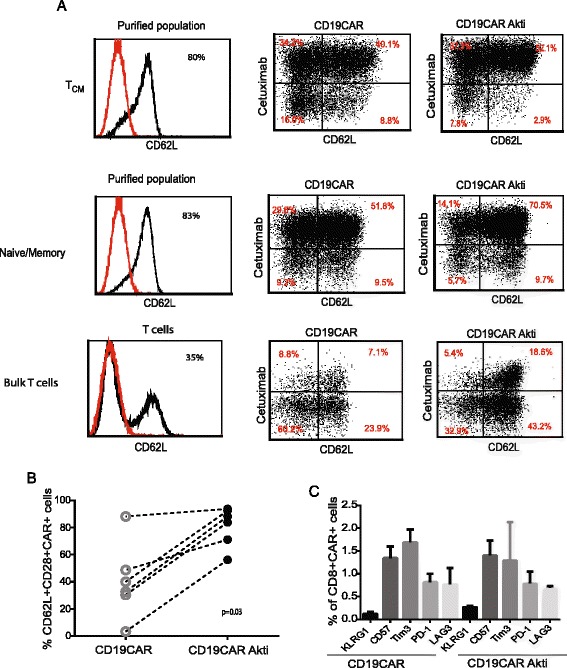
Akt inhibition promotes the generation of memory CD19CAR T cells from different T cell subsets. **a** Bulk T cells, purified TCM, and purified naïve/memory T cells were transduced with lentivirus encoding second generation CD19CAR vector and expanded in a medium containing 50U/L rhIL2, in the presence and absence of 1 μM Akt inhibitor for 17–21 days. Resultant CD19CAR T cells were stained with biotinylated Erbitux (cetuximab), followed by streptavidin-PE for CAR detection and antibodies against CD62L. Percentages of CAR + CD62L+ cells are depicted on the basis of the gating of isotype-stained cells. **b** Percentages of CD62L + CD28+ T cells after gating on CAR + CD8+ from six lines derived from two different donors are presented. **c** Mean percentages of immunoreactive cells + SEMs from six lines of two different donors are presented

CD3/CD28 bead stimulation and activation have been used for engineering CAR T cells to improve transduction efficiency and expansion to a therapeutic dose. However, the levels of stimulation are super-physiological, and over-stimulation possibly induces terminal differentiation and functional impairment of CAR T cells [24], which could be one reason for poor antitumor response observed in some patients. Our hypothesis was that Akti would decrease the magnitude of activation and therefore preserve memory-like T cells with improved potential for persistence and antitumor activity after adoptive transfer. Expanded T cells were analyzed for memory T cell characteristics. After 17 days of ex vivo culture of CD8 + CD19CAR T cells, expression of CD62L was 18.2% ± 7.1 in the Akti-untreated culture and 33.9% ± 11.3 in the Akti-treated counterparts (*N* = 4, *P* = 0.1). CD62L + CAR+ double positive cells were 34.9% ± 9.7 and 58.0% ± 9.7 (*N* = 4, *P* = 0.2) in the Akti-untreated culture and in the Akti-treated counterparts, respectively (Fig. 2a and b). Consistently, when gated CAR+ cells were analyzed, Akti-treated CD8 + CD19CAR T cells co-express higher levels of CD62L and CD28 as compared to the untreated CD19CAR T cells (50.3% ± 3.7 vs.10.3% ± 4.8) (*N* = 4, *P* = 0.1) (Fig. 2c and d), suggesting that Akti-expanded CD8 + CD19CAR T cells appear less differentiated and possess a memory phenotype. To further understand the levels of senescence and exhaustion of the CD8 + CD19CAR T cells, we analyzed expression of KLRG1, CD57, Tim3, PD1 and LAG3, which are known to be the hallmarks of exhaustion. Our data support that Akti did not increase the senescence of CD8 + CD19CAR T cells (Fig. 2e). We also measured and compared the relative telomere lengths. In line with the findings that Akti had no effects on proliferative capacity and senescence of the CD8 + CD19CAR, Akti-treated, purified CD8 + CD19CAR T cells derived from three donors maintained relatively equal telomere length (Fig. 2f) as compared to untreated CD8 + CD19CAR T cells (*N* = 3, *P* = 0.3). Although purified T_CM_ and naïve/memory T cells originally express high levels of CD62L (80%), after activation/lentiviral transduction and ex vivo expansion, CD62L expression decreased to 40–50%. Consistently, Akti addition preferentially retained CD62L expression on the cultured cells (Fig. 3a) to 50–80%. The same results were observed when unselected bulk T cells were tested. These studies demonstrate that manipulation of levels of Akt activation could prevent differentiation derived from CD3/CD28 activation/expansion and promote enhanced central memory T cell subsets. Consistent with engineered CD19CAR T cells from purified CD8+ T cells, CD28 + CD62L + CAR+ T cells derived from bulk PBMC, T_CM_ and naïve/memory T cells are significantly higher in the Akti treated conditions as compared to untreated counterparts (*N* = 6, *P* = 0.03) (Fig. 3b). However, exhaustive features remain the same, indicating that Akti prevents CAR T cell differentiation and does not induce exhaustion of CAR T cells (Fig. 3c).

### Akt inhibition does not dampen cell-mediated cytotoxicity but increases cytokine production

**Fig. 4:**
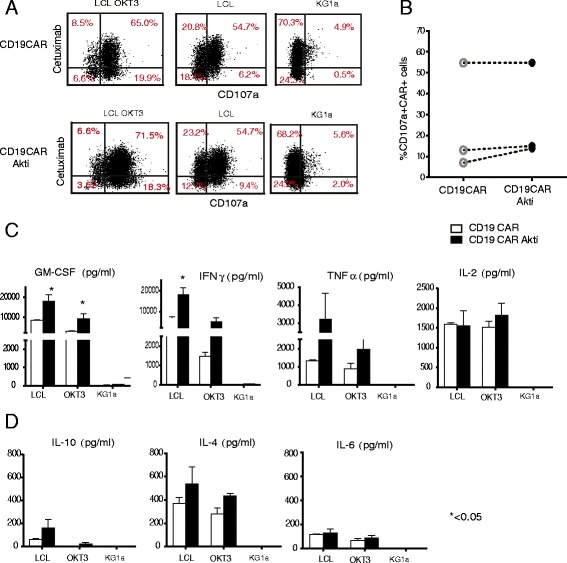
Akt inhibition does not dampen cell-mediated cytotoxicity but increases cytokine production of CD8 + CD19CAR T cells. After 21 days of in vitro expansion, **a** CD8 + CD19CAR T-cells with and without Akt inhibitor treatment were co-cultured with LCLs at a 1:1 ratio in medium containing Golgi plug and CD107a for six hours at 37 °C. KG1a cells were used as negative stimulator and LCL OKT3 as positive stimulator. Degranulation was determined using multicolor flow cytometry. Percentages of CAR + CD107a + T cells are presented. **b** Accumulated data from three different donors are presented. **c** CD8 + CD19CAR T cells with and without Akt inhibitor treatment were co-cultured with LCLs at a 1:1 ratio in medium for eighteen hours at 37 °C. Supernatants were harvested and cytokines were measured by a cytometric bead array. Means + SEMs of triplicates are depicted. KG1a cells were used as a negative stimulator and LCLOKT3 as positive stimulator

Akt activation appears to be essential for the development of effector function in activated CD8+ T cells [25] and is required for efficient adoptive therapy. To assess this functionality, we analyzed Akti-treated CD8 + CD19CAR T-cells for degranulation ability upon co-culture with different targets. We found equivalent levels of CD107a + cells in CD19CAR and Akti-treated CD19CAR T cells upon CD19+ tumor stimulation (24.9 ± 15 vs. 27.8 ± 13.4%, respectively) (Fig. 4a and b). Maximum degranulation remained the same as indicated by the degranulation ability after stimulation with LCL OKT3, which engages the entire T cell receptor (TCR), suggesting that the tumor-specific and intrinsic capacity of effector function was not affected by Akt inhibition. Cytokine production, another benchmark of effector function, was analyzed. Cytokines that were released into the supernatant after stimulator and responder co-culture were measured by cytometric bead array. Significantly higher levels of Th1 cytokines IFNγ (*P* < 0.05) and GM-CSF (*P* < 0.05) were produced by CD8 + CD19CAR T cells treated with Akti (Fig. 4c) upon CD19 antigen (LCL) stimulation, indicating a population with greater effector function. However, Th2 cytokines such as IL-10, IL-4, and IL-6 that hamper the induction of effective of T cell response were not significantly increased upon CD19 antigen stimulation (Fig. 4d).

### Akti-expanded CD19CAR T-cells appeared to exhibit greater anti-tumor efficacy and expansion in vivo

**Fig. 5:**
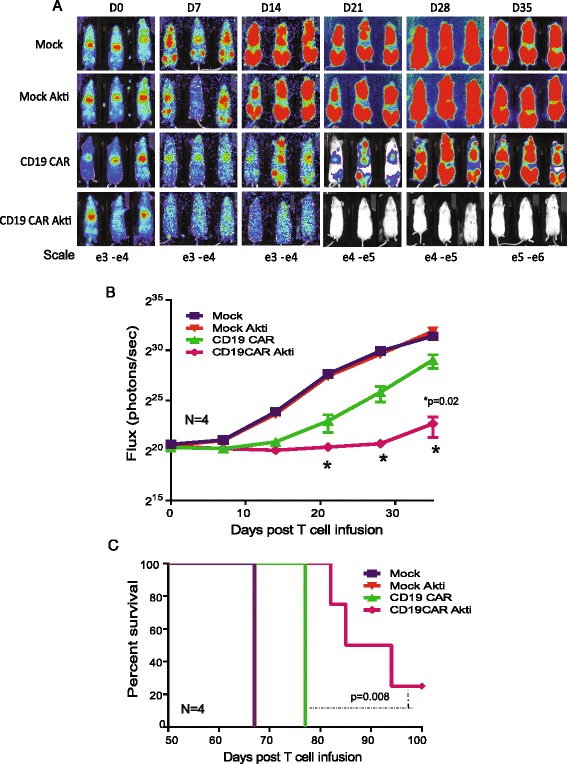
Akti-treated CD8 + CD19CAR T cells exhibit increased anti-tumor efficacy in vivo. 0.5 × 10^6^ acute lymphoid leukemic SupB15 cells engineered with GFP firefly luciferase (GFPffluc+) were inoculated into NSG mice intravenously (i.v) on day -5. After confirmation of tumor engraftment, 2 × 10^6^ expanded CD8 + CD19CAR+ T cells were adoptively transferred into tumor-bearing mice intravenously. Non-transduced mock cells from the same donor were used as controls. **a** Tumor signals were monitored by biophotonic tumor imaging and **b** the bioluminescence signal was measured as total photon flux normalized for exposure time and surface area and expressed in units of photons (p) per second per cm^2^ per steradian (sr). **c** Kaplan-Meier survival curve. *N* = 4 mice per group

**Fig. 6:**
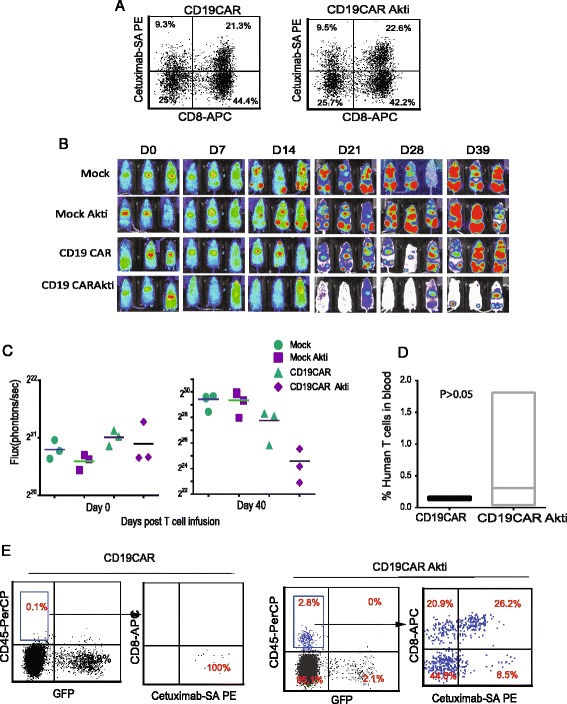
Akti-treated CD4 + CD19CAR+ and CD8 + CD19CAR T cells exhibit increased anti-tumor efficacy and persistence in vivo. PBMC from healthy donors were activated with CD3/CD28 Dynabeads and transduced the next day with CD19R (EQ):CD28:ζ/EGFRt lentivirus at an MOI of 3. The cultures were supplemented with 50 U/mL rhIL-2 and Akt inhibitor every 48 h for 21 days. Transduced CD19CAR T cells without Akt inhibitor treatment were used as control. Non-transduced mock T cells were used as another type of control. **a** Percentages of CD8 and CAR positive cells in the input population are presented. **b** Acute lymphoid leukemic 0.5 × 10^6^ SupB15 cells engineered with GFPffluc were inoculated into NSG mice (*N* = 3 mice per group) intravenously (i.v) on day -5. After confirmation of the tumor engraftment, 1 × 10^6^ expanded CD19CAR+ T cells were adoptively transferred into tumor-bearing mice intravenously. Tumor signals were monitored by Biophotonic tumor imaging and **c** the bioluminescence signal was measured as total photon flux normalized for exposure time and surface area and expressed in units of photons (p) per second per cm^2^ per steradian (sr). **d** Forty days post adoptive transfer, mice were euthanized, and blood were analyzed after staining with antibodies against human CD45, CD8 and cetuximab for CAR detection. Mean percentages of human CD45 positive and GFP (tumor) negative cells from three mice are presented. **e** CD8+ and CAR T cells gated on CD45 positive human T cells are depicted

**Fig. 7:**
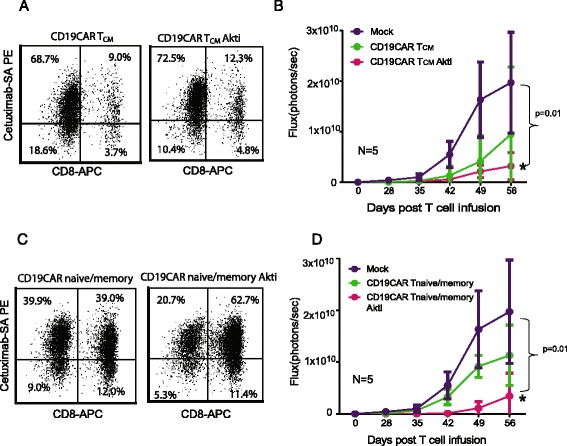
Akti-treated CD19CAR T_CM_ + and CD19CAR naïve/memory T cells exhibit greater anti-tumor efficacy in vivo. T_CM_ and naïve/memory T cells from healthy donors were isolated and activated with CD3/CD28 Dynabeads and transduced the next day with CD19R (EQ):CD28:ζ/EGFRt lentivirus at an MOI of 3. The cultures were supplemented with 50 U/mL rhIL-2 and Akt inhibitor every 48 h for 21 days. Transduced CD19CAR T cells without Akt inhibitor treatment were used as control. Non-transduced mock T cells were used as another type of control. Acute lymphoid leukemic 0.5 × 10^6^ SupB15 cells engineered with GFPffluc were inoculated into NSG mice (*N* = 5 mice per group) intravenously (i.v) on day -5. After confirmation of the tumor engraftment, 1 × 10^6^ expanded CD19CAR+ T cells were adoptively transferred into tumor-bearing mice intravenously. Tumor signals were monitored by Biophotonic tumor imaging and the bioluminescence signal was measured as total photon flux normalized for exposure time and surface area and expressed in units of photons (p) per second per cm^2^ per steradian (sr). **a** Percentages of CD8 and CAR positive cells in the input CD19CAR T_CM_ population are presented. **b** Tumor signals at different time points are presented. **c** Percentages of CD8 and CAR positive cells in the input CD19CAR naïve/memory population are presented. **d** Tumor signals at different time points are presented

The principal aim of the study was to determine whether Akti promotes the generation of CAR T cells with enhanced antitumor activity. Building on the memory-like phenotypic traits and improved cytokine production, we anticipated that Akti-expanded CD8 + CD19CAR T cells would possess enhanced anti-tumor activity once transferred into B cell leukemia tumor-bearing mice. To test the antitumor activity, we delivered a suboptimal (2 × 10^6^) dose of CD8 + CD19CAR T-cells intravenously into mice following systemic inoculation of human CD19+ leukemic cells. Tumor burden in the mice was monitored on a weekly basis, and Kaplan-Meier survival analysis was performed. Consistent with the in vitro observation, untreated CD8 + CD19CAR T cells induced transient tumor regression, but tumors re-progressed rapidly. In contrast, mice treated with an equal number of Akti-treated CD8 + CD19CAR exhibited significantly higher antitumor activity (*p* = 0.02) with significantly prolonged survival (*p* = 0.008) (Fig. 5). One mouse had no evidence of tumor 100 days from inoculation. We also tested the anti-tumor activity of bulk CD19 CAR-T cells containing CD4 T cells and CD8 T cells that were expanded in the presence of Akti and compared them with non-treated cells having the same proportion of CD4, CD8, and CAR subsets. We again observed what appeared to be more tumor regression when mice were treated with Akti-treated CD19CAR T cells than untreated counterpart (Fig. 6a, b, and c). Furthermore, 40 days post CAR T cell transfer, higher levels of human T cells and CAR T cells were detected in the mice that received Akti-treated CD19CAR T cells than that in the mice that received non-Akti-treated counterparts, although the results are not statistically significant (Fig. 6d). Percentages of CAR T cells in the harvested blood were similar to the input cells (~30%) (Fig. 6e), suggesting that Akti-treated CD19CAR T cells were proportionately expanded in vivo following adoptive transfer. To demonstrate Akti has similar impacts on different T cell subsets, propagated CD19CAR T cells derived from purified T_CM_ and naïve/memory T cells in the presence and absence of Akti were tested for their antitumor activity in vivo. Consistently, Akti-treated CD19CAR T_CM_ (Fig. 7a and b) and naïve/memory T cells (Fig. 7c and d) exhibited greater antitumor activity as compared with their untreated counterparts even though the CD4+ CAR T cells are dominant in the T_CM_ populations. To rule out the xeno-reactivity of infused Akti-treated CD19CAR T cells, mouse body weight, an indicator of GVHD, was measured. We observed no evidence of GVHD such as loss of body weight (Additional file 2: Figure S2) and hair loss, on day 28, when the highest active antitumor activity occurred.

## Discussion

Effective T cell therapy against cancer is dependent on the formation of long-lived memory T cells with the ability to self-renew and differentiate into potent effector cells. These goals have been approached by various means, including using defined central memory T cells as a starting population for gene modification, optimizing CAR design, and supplementing with cytokine cocktails during ex vivo expansion [8, 18, 26, 27]. All of these strategies are feasible, but validation before translating to a clinical platform is costly and time-consuming. There are several pharmacologic candidates capable of driving T cells toward the memory phenotype, such as those activating the Wnt/β-catenin pathway [14] and inhibiting m-TOR mediated protein translation [28]. However, the concentration required for significant inhibition of T cell differentiation might also lead to immunosuppressive and anti-proliferative activity [29, 30]. Our data support that addition of Akti at 1 μM during ex vivo expansion did not interfere with CD8 + CD19 CAR T cell proliferation and expansion. The same results were also observed in different T cell subsets such as bulk T cell, T_CM,_ and naïve/memory-derived CD19CAR T cells, indicating that this is a general feature of the Akti on T cell proliferation and expansion and therefore can be used for clinical manufacturing of therapeutic doses of CAR T cell products without the restriction of starting T cell populations. Our data indicated that Akti at 1 μM resulted in modest reduction (12%) of phosphorylated Akt in the CAR T cells. A ~5-fold increase in concentration is anticipated to further reduce phosphorylated Akti at Ser 473 [31]. However, careful titration of Akti during CAR T cell generation is necessary to maximize potency without suppressing T cell expansion and function. The optimal concentration of Akti may additionally vary among the various T cell subsets as starting population. Efficient CAR T cell therapy relies on potent effector function and in vivo persistence following adoptive transfer. Our studies demonstrated that manipulation of levels of Akt activation enhanced the antigen-specific effector function of CD19CAR T cells as indicated by elevated Th1 cytokines such as IFNγ and GM-CSF while preventing differentiation. Studies have shown that T cells with memory characteristics positively correlate with in vivo persistence and antitumor activity post adoptive transfer [32]. Despite no statistically significant differences as a result of variation among donors and a limited number of donors, our studies consistently demonstrated that Akti-treated CD19CAR T cells exhibited higher expression levels of CD62L and CD28 without expression of exhaustion phenotypes such as KLRG1, which are the traits required for efficient anti-tumor activity following adoptive transfer [33]. In further support, there did not appear to be a difference in telomere length between cultures with or without Akti. Upon transfer into CD19 tumor-bearing mice, Akti-treated CD8 + CD19CAR T cells efficiently inhibited tumor growth and induced long-term survival as a result of improved antitumor activity and/or expansion. The role of the Akt signaling pathway in CD4+ cells is more complicated and poorly understood than in CD8+ cells because of the interplay of regulatory versus conventional CD4+ T cells that contain different CD4+ T cell subsets. However, more tumor reduction in the mice treated with an Akti-inhibited mixture of CD4 + CD19CAR and CD8 + CD19 CAR T cells indicates the minor impact of Akti-treated Tregs on the overall therapeutic effects. This validates the use of Akti when both CD4+ and CD8+ T cells are included in the starting population for CAR T cell engineering and manufacturing. We also found that Akti-treated CAR T cells did not induce alloreactivity, an important safety consideration. Built on the pre-clinical studies [8–10, 34] demonstrating that T_CM_ and naive/ memory T cells possess characteristics that correlate with improved anti-tumor activity, current clinical trials have developed a clinical platform for purification, transduction, and expansion of CD19CAR T_CM_ for adoptive immunotherapy, with the goal of long-term CAR T cell disease surveillance [35]. Our current studies demonstrate that Akti does not inhibit T_CM_ and naïve/memory-derived CD19CAR T cell expansion but promotes the generation of memory CD19CAR T cells with greater antitumor activity and supports the use of Akti for further improved CAR T cell therapy in these advanced platforms. The underlying mechanisms may be related to changes in metabolic and transcriptional signatures characteristic of long-lived memory T cells that are reprogrammed by Akt inhibition [16, 25, 36]. Meanwhile, our data support that Akt inhibition has no impact on replicative senescence based on the lack of changes in relative telomere length and exhaustive features.

## Conclusion

Our data suggest that inhibition of Akt signaling during ex vivo priming, expansion, and culturing gives rise to a CD19CAR T cell population that possesses increased antitumor activity. These findings suggest that therapeutic modulation of Akt might be a strategy to augment antitumor immunity for adoptive CAR T cell therapy, which could easily be transitioned into the clinic with the availability of a pharmaceutical grade Akt inhibitor.

## Additional files

Additional file 1: Figure S1. Schematic of the CD19CAR-T2A-EGFRt. The CD19R:zeta DNA sequence (optimized by GeneArt) contains the chimeric antigen receptor (CAR) sequence consisting of the V_H_ and V_L_ gene segments (scFv) of the CD19-specific FMC63 monoclonal antibody (mAb); IgG4 hinge containing the 2 point mutations, L235E and N297Q in the CH2 portion (CD19R(EQ)); the CD28 costimulatory domain; signaling domains CD3 zeta; and the self-cleaving T2A and the truncated EGFR (EGFRt) sequences. (EPS 1663 kb)
Additional file 2: Figure S2. CD19CAR T cells expanded in the presence of Akti did not induce allo-reactivity in xenograft model. T cell subsets were isolated and activated with CD3/CD28 Dynabeads and transduced the next day with CD19R (EQ):CD28:zeta/EGFRt lentivirus at an MOI of 3. The cultures were supplemented with 50 U/mL rhIL-2 and Akt inhibitor every 48 h for 21 days. Acute lymphoid leukemic 0.5 × 10^6^ SupB15 cells engineered with GFPffluc were inoculated into NSG mice intravenously (i.v) on day -5. After confirmation of the tumor engraftment, 1 × 10^6^ expanded CD19CAR+ T cells were adoptively transferred into tumor-bearing mice intravenously. Transduced CD19CAR T cells without Akt inhibitor treatment were used as control. Non-transduced mock T cells were used as another type of control. Body weight was measured on day 28 post T cell infusion, when the most active antitumor activity occurred in CD19CAR Akti treated mice. Means ± SEMs are presented. Number of mice in each group is presented on the top of the bar. (EPS 1490 kb)

## Abbreviations

Akti: Akt inhibitor
CAR: Chimeric antigen receptor
CFSE: Carboxyfluorescein succinimidyl ester
EGFRt: Truncated human EGFR
GFPffluc: Green fluorescence firefly luciferase
LCL: Lymphoblastoid cell lines
T_CM_: Central memory T cells
